# The Relationship between Tumor Budding and Tumor Deposits in Patients with Stage III Colorectal Carcinoma

**DOI:** 10.3390/jcm13092583

**Published:** 2024-04-27

**Authors:** Zdenko Bilić, Mario Zovak, Goran Glavčić, Dubravka Mužina, Amir Ibukić, Andro Košec, Davor Tomas, Alma Demirović

**Affiliations:** 1 Department of Surgery, Sestre Milosrdnice University Hospital Center, 10 000 Zagreb, Croatia; zbilic84@gmail.com (Z.B.); mzovak73@gmail.com (M.Z.); glavcic.goran@gmail.com (G.G.); dubi.muzina@gmail.com (D.M.); ibukic@gmail.com (A.I.); 2 School of Medicine, University of Zagreb, 10 000 Zagreb, Croatia; andro.kosec@yahoo.com (A.K.); davor.tomas@kbcsm.hr (D.T.); 3 School of Dental Medicine, University of Zagreb, 10 000 Zagreb, Croatia; 4 Department of Otorhinolaryngology & Head and Neck Surgery, University Hospital Center Sestre Milosrdnice, 10 000 Zagreb, Croatia; 5 Department of Pathology and Cytology, Sestre Milosrdnice University Hospital Center, 10 000 Zagreb, Croatia

**Keywords:** colorectal carcinoma, tumor budding, tumor deposits, epithelial-mesenchymal transition

## Abstract

**Background/Objectives:** Recently, some new morphological features of colorectal cancer have been discovered as important prognostic factors; in this paper, we study the relationship between tumor budding (TB) and tumor deposits (TDs). **Methods:** The retrospective cohort study included 90 patients with pathohistologically confirmed stage III CRC who were treated with radical surgical resection. All hematoxylin and eosin (H and E)-stained slides from each patient were reviewed, and histological parameters were recorded. The samples were divided into two groups with similar sizes: a group without TDs (N = 51) and a control group with TDs (N = 39). The presence and TB grade were further analyzed in these groups and compared with other clinical and histological features. **Results:** The prevalence of TB in the investigated cohort was unexpectedly high (94.4%). Overall, there were 23 (25.6%) Bd1, 20 (22.2%) Bd2, and 47 (52.2%) Bd3 cases. The presence of TDs was significantly associated with a higher number of TB (*p* < 0.001, OR 16.3) and, consequently, with a higher TB grade (*p* = 0.004, OR 11.04). A higher TB grade (*p* = 0.001, HR 2.28; 95% CI 1.93–4.76) and a growing number of TDs (*p* = 0.014, HR 1.52; 95% CI 1.09–2.1) were statistically significantly associated with shorter survival. **Conclusions:** TDs appear more often in patients with higher TB grades in stage III CRC. A higher TB grade and a growing number of TDs were statistically significantly associated with shorter overall survival. These results could give additional emphasis to the importance of TB as an adverse prognostic factor since a strong relationship with TDs has been demonstrated.

## 1. Introduction

Colorectal carcinoma (CRC) ranks third in incidence in men and second in women, with about 1.9 million newly diagnosed cases and more than 900,000 deaths worldwide annually [1]. Determining the exact stage of the disease enables clinicians to adopt more reliable treatment algorithms. The assessment of CRC spread is performed with the TNM system, a globally recognized standard and the most widely used cancer staging system [2,3,4,5]. Despite this, it was proved insufficiently precise and unreliable in some clinical scenarios due to significant variation in final outcomes in patients with the same disease stage [6,7,8,9].

Some new morphological features of CRC have been detected lately as significant prognostic factors that describe the interaction of tumor tissue with the neighboring stroma and how it affects the potential for invasive tumor growth and metastasizing [8,9,10,11,12,13]. Some of these features studied in our research were tumor budding (TB) and tumor deposits (TDs).

TB, described in 20–40% of CRC cases [7,14], represents the local migration of a small group of dedifferentiated tumor cells (<5 cells) from the edge of the invasive cancer growth into the surrounding peritumoral stroma [15,16,17]. Precise data on TB prevalence in stage III CRC are lacking. A high TB grade is an independent prognostic marker and correlates with a poor prognosis [16,18,19]. TB is considered a morphological manifestation of epithelial–mesenchymal transition (EMT), where cancer cells lose elements of epithelial cells and take on mesenchymal cells’ characteristics, leading to increased invasiveness, propensity for migration, and resistance to apoptosis. These changes allow cells to separate from the primary tumor mass and form cell buds in the peritumor connective tissue [20,21,22,23,24].

TDs refer to the extramural focal accumulation of tumor cells in the peritumoral tissue, which has no continuity with the primary tumor mass and is not connected to the surrounding lymph nodes [25,26,27]. The incidence of TDs in CRC varies widely from 4% to 45% [27,28,29,30]. The definition of TDs has changed throughout history. In the TNM-8 classification, additional rules were introduced that exclude the presence of lymph node architecture and the identification of vascular or neural structures around the TDs, making a clear distinction between TDs and separate entities, i.e., lymphovascular invasion (LVI), extramural venous invasion (EMVI), and perineural invasion (PNI) [5]. It was shown that the presence of TDs correlates with an increased risk of local disease recurrence and, generally, a worse prognosis [8,10,25,27,31,32,33,34,35].

The origin of TDs was the subject of numerous studies, and the process of how TDs are formed is still unclear [30,32,34].

In our study, we hypothesized that TDs are more common in patients with stage III CRC with a more expressed TB grade. Thus, the general objective of this study was to analyze the presence of TDs and their association with the presence of TB in pathohistological samples obtained from patients with stage III CRC after radical surgical resection. As additional specific objectives, we aimed to determine the presence and TB grade in the pathohistological samples, to estimate the presence and the diameter of the largest TDs, to determine the possible association between the TB grade and the size of TDs, and finally to analyze the potential relationship between TB and TDs with clinicopathological parameters in patients with stage III CRC.

We believe that in stage III CRC, TDs could appear more frequently in patients with higher TB grades. While there has been some research looking at TB and TDs together [11], a further literature review found no studies specifically regarding the possible relationship between TB and TDs so far. Therefore, we consider this study desirable and necessary to fill the literature gap.

If the relationship between TB and TDs is confirmed, and considering that TDs have been particularly emphasized as an adverse prognostic factor in the two most recent TNM classifications under category ‘N1c’, it could also provide added weight to the importance of TB as an additional adverse prognostic factor. Additionally, it could be beneficial for stratifying patients into more precisely defined prognostic groups. Therefore, further research on this topic could lead to even more pronounced recommendations from professional societies regarding the management of cases with pronounced TB.

## 2. Materials and Methods

### 2.1. Study Design and Participants

The retrospective cohort study included 90 patients, treated with radical surgical resection at the Department of Surgery, University Hospital Center Sestre milosrdnice, Zagreb, Croatia, between 1 January 2009, and 31 December 2013, with pathohistologically confirmed stage III CRC. The study excluded rare histological subtypes, i.e., medullary, micropapillary, adenosquamous, undifferentiated, or mixed subtypes, and patients who underwent neoadjuvant chemoradiotherapy, patients with known metastatic disease at the time of surgery, or those who underwent an emergency surgery procedure.

Patients’ clinical data (age, gender, tumor location, overall survival) were retrieved from the patients’ data archive and the Croatian National Cancer Registry. The retrieval of archival tissue and patients’ clinical data was conducted while respecting all ethical permissions for this study. Ethical approvals were obtained from the Ethics committee of the Medical Faculty University of Zagreb (641-01/23-02/01), as well as the University Hospital Center Sestre milosrdnice (EP-2823/19-1).

### 2.2. Pathohistologically Assessment

All hematoxylin and eosin (H and E)-stained slides from each patient were reviewed, and histological parameters, including histological grade, pT, pN, TD presence, the diameter of the largest TD in mm, lymphovascular invasion (LVI), perineural invasion (PNI), extramural vascular invasion (EMVI), and TB grade, were recorded for each case. All patients acquired R0 resection with histological proof of negative resection margin. Histological parameters were assessed by two independent pathologists (DT and AD) blinded to the clinical data, and differences in interpretation were resolved by joint review.

**Figure 1:**
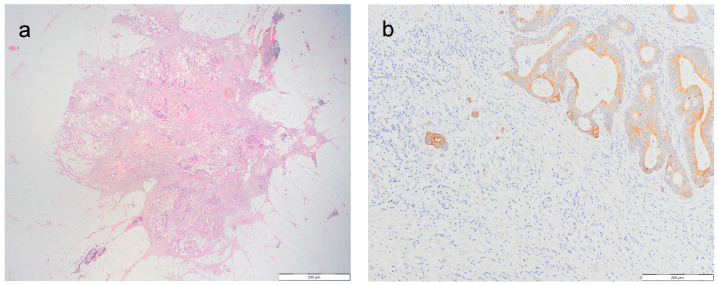

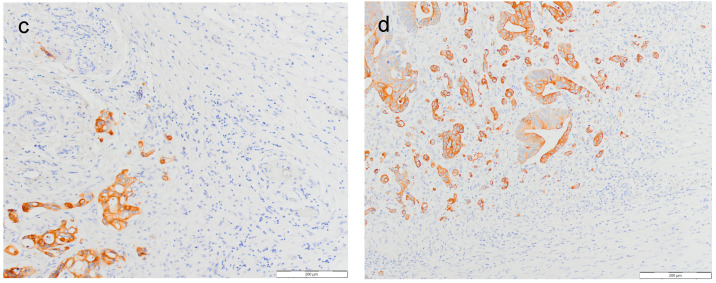
(**a**) Microscopic appearance of TDs, H and E ×20; (**b**) immunohistochemical reaction for CK-PAN: low-grade budding-Bd1 ×200; (**c**) immunohistochemical reaction for CK-PAN: intermediate-grade budding-Bd2 ×200; and (**d**) immunohistochemical reaction for CK-PAN: High-grade budding-Bd3 ×200.

Depending on the presence of TDs, pathohistological samples were divided into two similar sized groups: a group without TDs (N = 51) and a control group with TDs (N = 39). The presence of TDs was revised according to the TNM-8 classification [2] (Figure 1a). The overall number of TDs and the diameter of the largest TD were determined. The diameter of the largest TD was measured with a computerized digital system with an Olympus BX51 microscope (Olympus Corporation, Tokyo, Japan), QuickPHOTO CAMERA, version 3.0 (PROMICRA, Prague, Czech Republic), and an Olympus Camedia Camera C 5050 (Olympus Corporation, Tokyo, Japan). The presence and TB grade were further analyzed in these groups and compared with other clinical and histological features.

TB was assessed on the H and E sections in a single 20× objective with a 22 mm field diameter and divided by the normalization factor 1.210, based on a 22 mm eyepiece field diameter, to obtain the standardized bud count. An additional analysis was performed with immunohistochemical staining with PAN-CK (DAKO, clone MNF116) on the selected blocks where the highest number of buds was assessed on H and E sections. The TB grade was determined according to the recommendations of the International Tumor Budding Consensus Conference (ITBCC) [28]. Bud count was divided into three-tier grades: low-grade Bd1 (0–4 buds), intermediate-grade Bd2 (5–9 buds), and high-grade Bd3 (≥10 buds) (Figure 1b–d).

### 2.3. Statistical Analysis

Descriptive and analytical statistical methods were used in the processing of the results. The tested variables were presented with standard descriptive indicators. The analysis of the normality of the data distribution was checked with the Smirnov–Kolmogorov test. According to the results, parametric and non-parametric tests were further used. A statistical analysis of the relationship between TDs and TB was performed using the Mann–Whitney U test and binary logistic regression analysis. A multivariate Cox regression analysis and Kaplan–Meier survival curves were used in the survival analysis. Associations between other pathohistological variables were compared further using the χ^2^ test, and Pearson’s and Spearman’s correlation coefficients. All statistical tests were two-tailed. *p* values ≤ 0.05 were determined to be statistically significant. Statistical data processing was performed with the help of the MedCalc program (Version 11.2.1 © 1993–2010. MedCalc Software bvba Software, Mariakerke, Belgium), and the SPSS program (Version 22.0. Released in 2013. IBM SPSS Statistics for Windows, IBM Corp., Armonk, NY, USA).

## 3. Results

The distribution of demographic and histopathological variables and their associations with different TB grades were recorded (Table 1). There were 55 male and 35 female patients (male-female ratio of 1.6). The average age was 65.8 years, ranging from 35 to 85 years. The average age of men was 66 ± 10.4 years, with a median of 69 years, and the average age of women 66 ± 10.9 years, with a median of 66.2 years. The average duration of patient follow-up (median) was 57.5 months, ranging from 2 to 163 months.

The investigated cohort consisted of 90 patients (N = 90). There were 51 patients in the group without TDs (N = 51) and 39 patients in the control group with present TDs (N = 39). In the latter, the average number of TDs was 2.3 ± 1.7, and the average diameter of the largest measured TD was 5.6 ± 3.4 mm. The prevalence of TB in the investigated cohort was unexpectedly high (94.4%). Overall, there were 23 (25.6%) Bd1, 20 (22.2%) Bd2, and 47 (52.2%) Bd3 cases.

**Figure 2:**
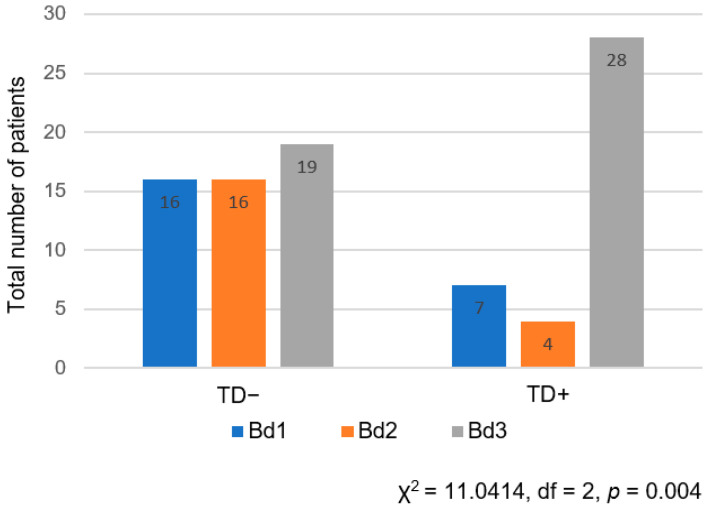
Distribution of TB grades regarding the presence of TDs in pathohistological samples.

A statistical analysis of the association between TDs and TB was performed using the Mann–Whitney U test, which showed that TD presence was significantly associated with a higher number of TB (*p* = 0.001). This finding was also confirmed in a binary logistic regression model with TDs as the dependent variable (*p* < 0.001, OR 16.3). Similar results were also obtained using the χ^2^ test, which also showed that the presence of TDs was significantly associated with a higher TB grade (χ^2^ = 11.0414, df = 2, *p* = 0.004) (Figure 2). No statistically significant difference was found in the distribution of TB grades between different subgroups of stage III (IIIA, IIIB, IIIC) (χ^2^ = 2.048, df = 4, *p* = 0.727).

**Figure 3:**
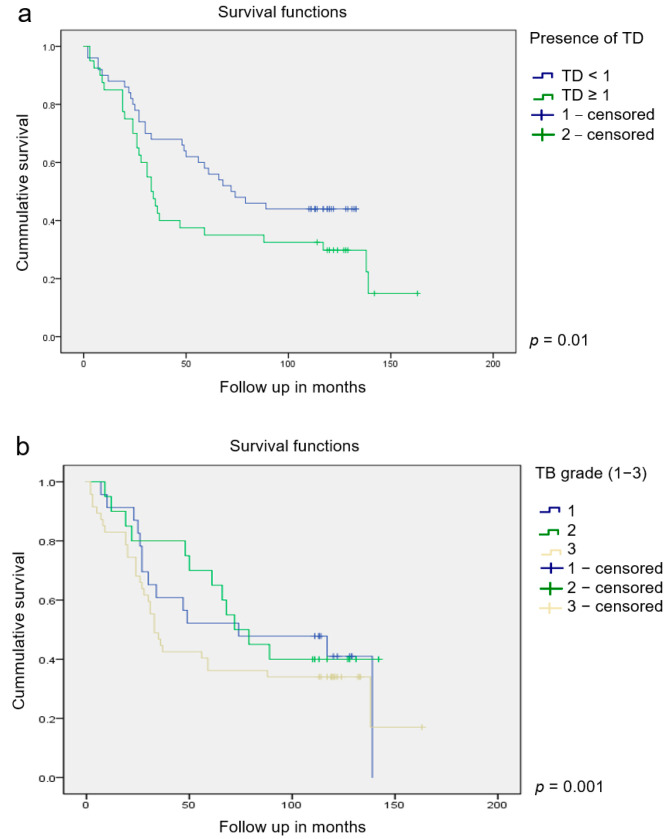
The Kaplan–Meier survival curves regarding (**a**) the presence of TDs and (**b**) TB grade.

The patient survival function regarding the TB grade (*p* = 0.01) and the presence of TDs (*p* = 0.001) is expressed by the Kaplan–Meier curve (Figure 3a,b).

A receiver operating curve (ROC) was performed to analyze the impact of the number of TB and TDs on survival, identifying a cut-off point associated with overall survival. The ROC analysis of the number of TDs and the relationship with overall survival showed that the cut-off value, expressed with the help of the Youden index (0.5586), where the specificity and sensitivity are the highest, equals 1. Sensitivity was 80% (95% CI, 44.4–97.5%), and specificity was 75.86% (95% CI, 56.5–89.7%).

**Figure 4:**
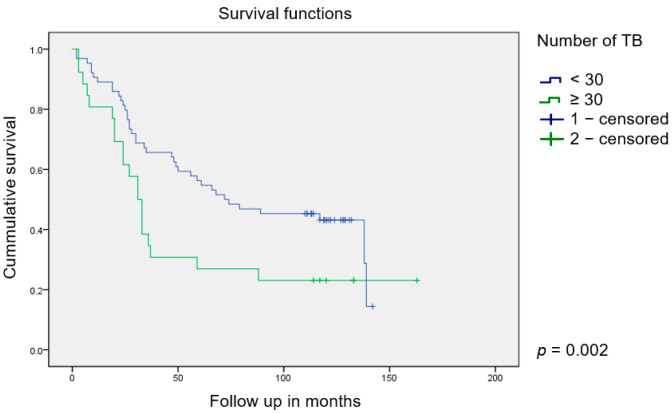
The Kaplan–Meier survival curves regarding the number of tumor buds (cut-off value = 30) and patient’s survival.

Further, the ROC analysis of the number of TB and the relationship with overall survival showed that the cut-off value, expressed with the help of the Youden index (0.1886), at which the specificity and sensitivity are the highest, equals 30. Sensitivity was 84.37% (95% CI, 67.2–94.7%), and specificity was 34.48% (95% CI, 22.5–48.1%). Regarding the established cut-off value of 30 TB, which was determined by the above statistical analysis, the association between the number of TB and patient survival was shown using the Kaplan–Meier survival curve (*p* = 0.002) (Figure 4).

The multivariate Cox regression analysis showed that a higher TB grade (*p* = 0.001, HR 2.28; 95% CI 1.93–4.76) and a growing number of TDs (*p* = 0.014, HR 1.52; 95% CI 1.09–2.1) were statistically significantly associated with shorter overall survival. It was also shown that the patient’s age, T, N category, and a higher TNM stage, EMVI, and LVI were statistically significantly associated with shorter survival (Table 2). The Pearson’s correlation coefficient for continuous variables showed that the TD diameter correlates with the number of TB (r = 0.366, *p* = 0.022).

## 4. Discussion

TB, as a suspicious morphological manifestation of EMT, represents the local migration of a small group of dedifferentiated tumor cells (<5 cells) from the edge of the invasive cancer growth into the surrounding peritumoral stroma. TDs, as the relatively new “N1c” category [4,5], represent extramural focal clusters of tumor cells located in peritumoral tissue and could represent a discontinuous pattern of tumor growth [30,36]. Although the processes by which TB and TDs are formed have not been fully clarified, the consequences of their presence in tumor tissues, especially in CRC, are well known.

The research from Landau et al. proved that TDs and high-grade TB are the most important independent predictors of tumor recurrence in stage III CRC [11]. Some other research has shown that patients with present TDs (N1c) have a lower disease-free survival rate than patients without TDs, even in the presence of lymph node metastases (N+). It was also proved that the presence of TDs in patients in stage II is associated with a higher incidence of local disease recurrence compared to patients in stage III without proven TDs [30,33,34].

Our study evaluated and compared the presence of TB and TDs in stage III CRC, demonstrating the connection between TDs and TB presence, which has not been described in the literature so far.

The origin of TDs was the subject of numerous studies, and the process by which TDs are formed is still not fully understood [25,26,27,29,30,37]. Regarding the definition of TDs, some additional rules were recently introduced through the TNM-8 classification [5]. Those rules exclude the presence of lymph node architecture and the identification of vascular or neural structures around the TDs, making a clear distinction between TDs and separate entities, i.e., lymph nodes, LVI, EMVI, and PNI [26,38]. It is considered that TDs do not arise directly via lymphogenic, capillary, or perineural pathways.

Our research has shown that in stage III CRC, TDs appear more frequently in patients with higher TB grades. The correlation observed between the presence of TDs, their total count, and the TB grade may imply that during the initial stages of tumor progression, the direct dissemination of dedifferentiated budding tumor cells into the surrounding stroma through EMT mechanisms is more plausible. Conversely, the likelihood of indirect spread via lymphogenic or perineural routes seems diminished. This suggests a potential interconnectedness in the developmental trajectory of TB and TDs, indicating that they could be part of a similar process. Even though statistical correlation does not necessarily imply a direct pathophysiological connection, it can still provide a foundation for future research aiming to uncover such associations.

These results could also give additional emphasis on the importance of TB as an adverse prognostic factor in stage III CRC, since a strong relationship with TDs has been proven; after many years of attempting to formulate a definition, TDs have now found their place in the two latest TNM classifications in the “N1c” category.

The deficiencies in the standardization of TB counting have limited its reporting in routine clinical practice. For this reason, data on the TB prevalence in CRC vary across different literature sources describing TB prevalence in 20–40% of CRC cases, regardless of the disease stage [7,11,14]. In our work, we showed that the prevalence of TB in stage III CRC is as high as 94.4%, which could be partially explained by the advanced stage of the disease. As we have not come across existing data regarding the prevalence of TB in stage III CRC thus far, we believe that the prevalence information obtained in our study could offer valuable insights.

Several systematization methods of an objective presentation of the TB grade have been published so far [28,39,40,41,42,43,44,45]. Our study determined the TB grade according to the recommendations of the International Tumor Budding Consensus Conference (ITBCC) in 2016, where the bud count was divided into three-tier grades: low-grade Bd1 (0–4 buds), intermediate-grade Bd2 (5–9 buds), and high-grade Bd3 (≥10 buds) [28].

Since its introduction in 2016, TB reporting according to the recommendations from ITBCC has been incorporated in many international clinical guidelines and protocols, such as the College of American Pathologists (CAP), the National Comprehensive Cancer Network (NCCN), and International Collaboration on Cancer Reporting (ICCR) [46,47,48,49].

According to the ITBCC statements with the unanimous agreement rate, TB is an independent predictor of lymph node metastasis in pT1 CRC, an independent predictor of survival in stage II CRC, and it should be taken into account along with other clinicopathological features within a multidisciplinary setting [28,50].

At least three different clinical scenarios have been identified where a TB assessment can offer valuable prognostic information. The most prominent are using TB assessments in pT1 CRC to influence clinical decisions regarding potential surgical resection versus a conservative approach, in stage II CRC to serve as one of the high-risk guiding treatments towards adjuvant chemotherapy, and to evaluate TB in biopsy samples, which may have significant clinical implications, particularly in the preoperative management of patients with rectal cancer, where neoadjuvant therapy could be considered [39,51].

As the reporting of TB became more widespread over time, new issues have emerged, prompting additional clarifications that have been extensively discussed in various sources in the literature. The importance of TB reporting has been subsequently confirmed through validation in numerous clinical research studies. Some of the new topics addressed in research papers following the ITBCC include the reproducibility of TB assessment, the development of automated algorithms for TB detection using the latest technologies, and the reevaluation of the ideal cut-off value, whether through a binary classification system or a three-tier classification system, for detecting points significantly affecting overall survival in most of the clinical scenarios [52,53,54,55,56,57,58].

In our study, given the relatively small cohort of patients, we established the cut-off through the receiver operating curve (ROC) analysis, identifying the value of 30 buds as the cut-off point significantly affecting overall survival. If a more extensive patient selection, encompassing all disease stages, was considered, the identified cut-off value would likely be smaller. It might be closer to the number of 10 buds, which is often used in the literature as an established cut-off value, especially in a two-tier classification system [45,59]

The positive and strong correlation between the diameter of the largest measured TD and the TB grade is likely attributed to the simultaneous growth period of TDs. It could be possible that the developmental timeframe during which TDs increase in dimension aligns with the time when the number of TB, and consequently, the TB grade, undergoes a similar progression.

The lack of a connection between the TB grade and the various TNM stages and subgroups (IIIA–C) in our study can likely be attributed to the homogeneity of the subjects, all of whom are in stage III. Additionally, the relatively small sample size might contribute to the challenge of identifying significant associations.

Considering the relatively small sample size and the fact that all subjects were in stage III, we nevertheless proved unequivocally that the presence and growing number of TDs and a higher TB grade were statistically significantly associated with shorter survival, which has been proven by numerous studies on a larger sample size and including all disease stages [13,26,29,30,37,40,45,60,61,62,63].

Discovering the possible link between TD development and the TB process in CRC patients could be beneficial for stratifying patients into more precise prognostic groups. Also, a better understanding of the TD formation could serve as a potential target in future investigations of novel therapeutic options.

Our study faces several limitations, with the primary constraint being its retrospective nature and reliance on a relatively small sample size obtained from a single institution. To enhance the robustness and generalizability of findings, future investigations should prioritize a multicenter approach for validation, incorporating a significantly larger sample size.

## 5. Conclusions

TDs appear more often in patients with higher TB grades in stage III CRC. The diameter of the largest TD positively correlates with the total number of tumor buds. A higher TB grade and a growing number of TDs were statistically significantly associated with shorter overall survival. These results could give additional emphasis to the importance of TB as an adverse prognostic factor, since a strong relationship with TDs has been demonstrated. Further research is needed to focus on multicenter validation with a larger sample size, comparing the obtained results with other results and also among different TNM stages.

## Figures and Tables

**Table 1:** Clinicopathological data of patients with stage III colorectal carcinoma.

Variable	Total (N = 90)	%	TB Grade						*p* *
			Bd1 (N = 23)	%	Bd2 (N = 20)	%	Bd3 (N = 47)	%	
Age									
<65	36	40.0%	9	39.1%	10	50.0%	17	36.2%	0.689
≥65	54	60.0%	14	60.9%	10	50.0%	30	63.8%	
Mean (±SD)	65.8 (±10.6)								
Gender	90	100.0%							
Male	55	61.1%	15	65.2%	14	70.0%	26	55.3%	0.314
Female	35	38.9%	8	37.8%	6	30.0%	21	44.7%	
Tumor location									
Right side	21	23.3%	6	26.1%	2	10.0%	13	27.7%	0.67
Left side	35	38.9%	10	43.5%	9	45.0%	16	34.0%	
Rectum	34	37.8%	7	30.4%	9	45.0%	18	38.3%	
Histological grade									
Low grade	73	81.0%	17	73.9%	17	85.0%	39	83.0%	0.465
High grade	17	19.0%	6	26.1%	3	15.0%	8	17.0%	
TD									
Presence (TD+)	39	43.3%	7	30.4%	4	20.0%	28	59.6%	0.002
Absence (TD−)	51	56.7%	16	69.6%	16	80.0%	19	40.4%	
TB									
Presence	85	94.4%	-	-	-	-	-	-	1
Mean No. (±SD)	22.5 (±24.2)		-	-	-	-	-	-	
Range	0–138								
Bd1	23	25.6%	-	-	-	-	-	-	
Bd2	20	22.2%	-	-	-	-	-	-	
Bd3	47	52.2%	-	-	-	-	-	-	
pT									
T1	1	1.1%	1	4.3%	0	0%	0	0%	0.214
T2	7	7.8%	1	4.3%	1	5.0%	5	10.6%	
T3	78	86.7%	18	78.3%	19	95.0%	41	87.2%	
T4	4	4.4%	3	13.1%	0	0%	1	2.2%	
pN									
N1	58	64.4%	14	60.9%	11	55.0%	33	70.2%	0.318
N2	32	45.6%	9	39.1%	9	45.0%	14	29.8%	
TNM Stage									
IIIA	9	10.0%	2	8.7%	1	5.0%	5	10.6%	0.219
IIIB	69	76.7%	16	69.6%	17	85.0%	36	76.6%	
IIIC	12	13.3%	5	21.7%	2	10.0%	6	12.8%	

* The Spearman rank correlation coefficient.

**Table 2:** Multivariate Cox regression analysis of clinical and pathohistological variables on patients’ overall survival.

Variables	Hazard Ratio	95% Confidence Interval	*p*-Value
Age, years, <65 vs. ≥65	1.31	1.1–1.08	0.001
Gender, female vs. male	0.281	0.27–1.56	0.092
Histological grade, LG vs. HG	0.9	0.45–1.82	0.107
T1, T2 vs. T3, T4	3.5	3.32–37.4	0.002
N1 vs. N2	8.55	2.65–8.64	0.009
TNM, IIIA vs. IIIB, IIIC	3.2	1.13–4.09	0.016
EMVI− vs. EMVI+	1.92	1.417–8.85	0.016
LVI− vs. LVI+	1.34	1.148–1.76	0.049
PNI− vs. PNI+	0.49	0.27–0.89	0.980
TD− vs. TD+	2.1	1.9–2.92	0.010
Number of TB, <30 vs. ≥30	1.9	1.36–3.74	0.002
TB grade, Bd1 vs. Bd2, Bd3	2.28	1.93–4.76	0.001

Abbreviations: LG—low grade, HG—high grade, EMVI—Extramural vascular invasion, LVI—Lymphovascular invasion, PNI—Perineural invasion, TD—Tumor deposits, TB—Tumor budding.

## Data Availability

The data contributing to this study’s findings can be obtained from the corresponding author upon reasonable request.

## References

[1] Sung H., Ferlay J., Siegel R.L., Laversanne M., Soerjomataram I., Jemal A., Bray F. (2021). Global Cancer Statistics 2020: GLOBOCAN Estimates of Incidence and Mortality Worldwide for 36 Cancers in 185 Countries. CA Cancer J. Clin..

[2] Sobin L., Wittekind C. (1997). International Union against Cancer (UICC). TNM Classification of Malignant Tumours.

[3] Sobin L., Wittekind C. (2002). International Union against Cancer (UICC). TNM Classification of Malignant Tumours.

[4] Sobin L., Gospodarowicz M., Wittekind C. (2009). International Union against Cancer (UICC). TNM Classification of Malignant Tumours.

[5] Brierley J., Gospodarowicz M., Wittekind C. (2017). International Union against Cancer (UICC). TNM Classification of Malignant Tumours.

[6] Burke H.B. (2004). Outcome Prediction and the Future of the TNM Staging System. JNCI J. Natl. Cancer Inst..

[7] Rogers A.C., Winter D.C., Heeney A., Gibbons D., Lugli A., Puppa G., Sheahan K. (2016). Systematic Review and Meta-Analysis of the Impact of Tumour Budding in Colorectal Cancer. Br. J. Cancer.

[8] Puppa G., Sonzogni A., Colombari R., Pelosi G. (2010). TNM Staging System of Colorectal Carcinoma: A Critical Appraisal of Challenging Issues. Arch. Pathol. Lab. Med..

[9] Maguire A., Sheahan K. (2014). Controversies in the Pathological Assessment of Colorectal Cancer. World J. Gastroenterol..

[10] Athanasakis E., Xenaki S., Venianaki M., Chalkiadakis G., Chrysos E. (2018). Newly Recognized Extratumoral Features of Colorectal Cancer Challenge the Current Tumor-Node-Metastasis Staging System. Ann. Gastroenterol..

[11] Landau M.A., Zhu B., Akwuole F.N., Pai R.K. (2019). Histopathological Predictors of Recurrence in Stage III Colon Cancer: Reappraisal of Tumor Deposits and Tumor Budding Using AJCC8 Criteria. Int. J. Surg. Pathol..

[12] Demirović A., Krušlin B. (2017). Recommendations for Histopathology Report of Colorectal Carcinoma Specimens. Rad. Hrvat. Akad. Znan. I Umjet..

[13] Riffet M., Dupont B., Faisant M., Cerasuolo D., Menahem B., Alves A., Dubois F., Levallet G., Bazille C. (2023). New Histoprognostic Factors to Consider for the Staging of Colon Cancers: Tumor Deposits, Invasive Tumor Infiltration and High-Grade Budding. Int. J. Mol. Sci..

[14] Zlobec I., Lugli A., Baker K., Roth S., Minoo P., Hayashi S., Terracciano L., Jass J.R. (2007). Role of APAF-1, E-cadherin and Peritumoural Lymphocytic Infiltration in Tumour Budding in Colorectal Cancer. J. Pathol..

[15] Hase K., Shatney C., Johnson D., Trollope M., Vierra M. (1993). Prognostic Value of Tumor “Budding” in Patients with Colorectal Cancer. Dis. Colon. Rectum.

[16] Ueno H., Mochizuki H., Hashiguchi Y., Hatsuse K., Fujimoto H., Hase K. (2004). Predictors of Extrahepatic Recurrence after Resection of Colorectal Liver Metastases. Br. J. Surg..

[17] Lugli A., Karamitopoulou E., Zlobec I. (2012). Tumour Budding: A Promising Parameter in Colorectal Cancer. Br. J. Cancer.

[18] Ueno H., Murphy J., Jass J.R., Mochizuki H., Talbot I.C. (2002). Tumour ‘budding’ as an Index to Estimate the Potential of Aggressiveness in Rectal Cancer. Histopathology.

[19] Berg K.B., Schaeffer D.F. (2018). Tumor Budding as a Standardized Parameter in Gastrointestinal Carcinomas: More than Just the Colon. Mod. Pathol..

[20] Thiery J.P., Acloque H., Huang R.Y.J., Nieto M.A. (2009). Epithelial-Mesenchymal Transitions in Development and Disease. Cell.

[21] Hanahan D., Weinberg R.A. (2011). Hallmarks of Cancer: The Next Generation. Cell.

[22] Li H., Xu F., Li S., Zhong A., Meng X., Lai M. (2016). The Tumor Microenvironment: An Irreplaceable Element of Tumor Budding and Epithelial-Mesenchymal Transition-Mediated Cancer Metastasis. Cell Adhes. Migr..

[23] Priya R., Yap A.S. (2015). Making a Choice: How Cadherin Switching Controls Cell Migration. Dev. Cell.

[24] Mashita N., Yamada S., Nakayama G., Tanaka C., Iwata N., Kanda M., Kobayashi D., Fujii T., Sugimoto H., Koike M. (2014). Epithelial to Mesenchymal Transition Might Be Induced via CD44 Isoform Switching in Colorectal Cancer. J. Surg. Oncol..

[25] Yabata E., Udagawa M., Okamoto H. (2014). Effect of Tumor Deposits on Overall Survival in Colorectal Cancer Patients with Regional Lymph Node Metastases. J. Rural. Med..

[26] Lord A.C., D’Souza N., Pucher P.H., Moran B.J., Abulafi A.M., Wotherspoon A., Rasheed S., Brown G. (2017). Significance of Extranodal Tumour Deposits in Colorectal Cancer: A Systematic Review and Meta-Analysis. Eur. J. Cancer.

[27] Ueno H., Mochizuki H., Hashiguchi Y., Ishiguro M., Miyoshi M., Kajiwara Y., Sato T., Shimazaki H., Hase K. (2007). Extramural Cancer Deposits Without Nodal Structure in Colorectal Cancer: Optimal Categorization for Prognostic Staging. Am. J. Clin. Pathol..

[28] Lugli A., Kirsch R., Ajioka Y., Bosman F., Cathomas G., Dawson H., El Zimaity H., Fléjou J.-F., Hansen T.P., Hartmann A. (2017). Recommendations for Reporting Tumor Budding in Colorectal Cancer Based on the International Tumor Budding Consensus Conference (ITBCC). Mod. Pathol..

[29] Nagtegaal I.D., Quirke P. (2007). Colorectal Tumour Deposits in the Mesorectum and Pericolon; a Critical Review. Histopathology.

[30] Jin M., Roth R., Rock J.B., Washington M.K., Lehman A., Frankel W.L. (2015). The Impact of Tumor Deposits on Colonic Adenocarcinoma AJCC TNM Staging and Outcome. Am. J. Surg. Pathol..

[31] Ueno H., Mochizuki H., Tamakuma S. (1998). Prognostic Significance of Extranodal Microscopic Foci Discontinuous with Primary Lesion in Rectal Cancer. Dis. Colon. Rectum.

[32] Prabhudesai A., Arif S., Finlayson C.J., Kumar D. (2003). Impact of Microscopic Extranodal Tumor Deposits on the Outcome of Patients with Rectal Cancer. Dis. Colon. Rectum.

[33] Bouquot M., Creavin B., Goasguen N., Chafai N., Tiret E., André T., Flejou J.-F., Parc Y., Lefevre J.H., Svrcek M. (2018). Prognostic Value and Characteristics of N1c Colorectal Cancer. Color. Dis..

[34] Belt E.J.T., Van Stijn M.F.M., Bril H., De Lange-de Klerk E.S.M., Meijer G.A., Meijer S., Stockmann H.B.A.C. (2010). Lymph Node Negative Colorectal Cancers with Isolated Tumor Deposits Should Be Classified and Treated As Stage III. Ann. Surg. Oncol..

[35] Gopal P., Lu P., Ayers G.D., Herline A.J., Washington M.K. (2014). Tumor Deposits in Rectal Adenocarcinoma after Neoadjuvant Chemoradiation Are Associated with Poor Prognosis. Mod. Pathol..

[36] Kim H.J., Choi G. (2019). Clinical Implications of Lymph Node Metastasis in Colorectal Cancer: Current Status and Future Perspectives. Ann. Coloproctol..

[37] Zhang L.-N., Xiao W.-W., Xi S.-Y., OuYang P.-Y., You K.-Y., Zeng Z.-F., Ding P.-R., Zhang H.-Z., Pan Z.-Z., Xu R.-H. (2016). Tumor Deposits: Markers of Poor Prognosis in Patients with Locally Advanced Rectal Cancer Following Neoadjuvant Chemoradiotherapy. Oncotarget.

[38] Kim H., Rehman A., Chung Y., Yi K., Wi Y.C., Kim Y., Jang K., Jang S.M., Paik S.S. (2016). Clinicopathologic Significance of Extranodal Tumor Extension in Colorectal Adenocarcinoma with Regional Lymph Node Metastasis. Gastroenterol. Res. Pract..

[39] Koelzer V.H., Canonica K., Dawson H., Sokol L., Karamitopoulou-Diamantis E., Lugli A., Zlobec I. (2016). Phenotyping of Tumor-Associated Macrophages in Colorectal Cancer: Impact on Single Cell Invasion (Tumor Budding) and Clinicopathological Outcome. OncoImmunology.

[40] Van Wyk H.C., Park J.H., Edwards J., Horgan P.G., McMillan D.C., Going J.J. (2016). The Relationship between Tumour Budding, the Tumour Microenvironment and Survival in Patients with Primary Operable Colorectal Cancer. Br. J. Cancer.

[41] Zlobec I., Lugli A. (2010). Epithelial Mesenchymal Transition and Tumor Budding in Aggressive Colorectal Cancer: Tumor Budding as Oncotarget. Oncotarget.

[42] Prall F. (2007). Tumour Budding in Colorectal Carcinoma. Histopathology.

[43] Shinto E., Mochizuki H., Ueno H., Matsubara O., Jass J.R. (2005). A Novel Classification of Tumour Budding in Colorectal Cancer Based on the Presence of Cytoplasmic Pseudo-fragments around Budding Foci. Histopathology.

[44] Mitrovic B., Schaeffer D.F., Riddell R.H., Kirsch R. (2012). Tumor Budding in Colorectal Carcinoma: Time to Take Notice. Mod. Pathol..

[45] Graham R.P., Vierkant R.A., Tillmans L.S., Wang A.H., Laird P.W., Weisenberger D.J., Lynch C.F., French A.J., Slager S.L., Raissian Y. (2015). Tumor Budding in Colorectal Carcinoma: Confirmation of Prognostic Significance and Histologic Cutoff in a Population-Based Cohort. Am. J. Surg. Pathol..

[46] Kakar S., Chanjuan S., Mariana B., Driman D., Fitzgibbons P., Frankel W., Hill K., Jessup J., Krasinskas A., Washington M. (2023). Protocol for the Examination of Specimens from Patients with Primary Carcinomaof the Colon and Rectum. https://documents.cap.org/protocols/ColoRectal_4.3.0.0.REL_CAPCP.pdf.

[47] Loughrey M.B., Webster F., Arends M.J., Brown I., Burgart L.J., Cunningham C., Flejou J.-F., Kakar S., Kirsch R., Kojima M. (2022). Dataset for Pathology Reporting of Colorectal Cancer: Recommendations from the International Collaboration on Cancer Reporting (ICCR). Ann. Surg..

[48] Benson A., Venook A., Adam M., Chen Y., Ciombor K., Cohen S., Cooper H., Deming D., National Comprehensive Cancer Network Colon Cancer Version 1.2024—January 29, 2024: NCCN Clinical Practice Guidelines in Oncology. https://www.nccn.org/professionals/physician_gls/pdf/colon.pdf.

[49] Benson A., Venook A., Adam M., Chen Y., Ciombor K., Cohen S., Cooper H., Deming D., National Comprehensive Cancer Network Rectal Cancer Version 1.2024—January 29, 2024: NCCN Clinical Practice Guidelines in Oncology. https://www.nccn.org/professionals/physician_gls/pdf/rectal.pdf.

[50] Zlobec I., Berger M.D., Lugli A. (2020). Tumour Budding and Its Clinical Implications in Gastrointestinal Cancers. Br. J. Cancer.

[51] Dawson H., Blank A., Zlobec I., Lugli A. (2019). Potential Clinical Scenarios of Tumour Budding in Colorectal Cancer. Acta Gastroenterol. Belg..

[52] Ueno H., Ishiguro M., Nakatani E., Ishikawa T., Uetake H., Matsuda C., Nakamoto Y., Kotake M., Kurachi K., Egawa T. (2019). Prospective Multicenter Study on the Prognostic and Predictive Impact of Tumor Budding in Stage II Colon Cancer: Results from the SACURA Trial. J. Clin. Oncol..

[53] Haddad T.S., Lugli A., Aherne S., Barresi V., Terris B., Bokhorst J.-M., Brockmoeller S.F., Cuatrecasas M., Simmer F., El-Zimaity H. (2021). Improving Tumor Budding Reporting in Colorectal Cancer: A Delphi Consensus Study. Virchows Arch..

[54] Koelzer V.H., Assarzadegan N., Dawson H., Mitrovic B., Grin A., Messenger D.E., Kirsch R., Riddell R.H., Lugli A., Zlobec I. (2017). Cytokeratin-based Assessment of Tumour Budding in Colorectal Cancer: Analysis in Stage II Patients and Prospective Diagnostic Experience. J. Pathol. Clin. Res..

[55] Luo Y.-H., Yan Z.-C., Liu J.-Y., Li X.-Y., Yang M., Fan J., Huang B., Ma C.-G., Chang X.-N., Nie X. (2024). Association of Tumor Budding with Clinicopathological Features and Prognostic Value in Stage III-IV Colorectal Cancer. World J. Gastroenterol..

[56] Wang K., He H., Lin Y., Zhang Y., Chen J., Hu J., He X. (2024). A New Clinical Model for Predicting Lymph Node Metastasis in T1 Colorectal Cancer. Int. J. Color. Dis..

[57] Kamall G.H., Ulusoy C., Nikolovski A., Kamall S. (2023). Tumour Budding—An Additional Prognostic Factor in Colorectal Cancer Survival. Pol. J. Pathol..

[58] Cyr D.P., Pun C., Shivji S., Mitrovic B., Duan K., Tomin R., Sari A., Brar A., Zerhouni S., Brar M.S. (2024). Tumor Budding Assessment in Colorectal Carcinoma: Normalization Revisited. Am. J. Surg. Pathol..

[59] Jurescu A., Dema A., Văduva A., Gheju A., Vița O., Barna R., Lăzureanu C., Cornianu M., Tăban S., Duță C. (2021). Poorly Differentiated Clusters and Tumor Budding Are Important Prognostic Factors in Colorectal Carcinomas. Bosn. J. Basic. Med. Sci..

[60] Lino-Silva L.S., Salcedo-Hernández R.A., Gamboa-Domínguez A. (2018). Tumour Budding in Rectal Cancer. A Comprehensive Review. Contemp. Oncol..

[61] Krištić A., Aralica G., Demirović A., Krušlin B. (2018). Pathohistologic Prognostic and Predictive Parameters in ColorectalcancerTumor Budding. Rad. Hrvat. Akad. Znan. I Umjet..

[62] Mirkin K.A., Kulaylat A.S., Hollenbeak C.S., Messaris E. (2018). Prognostic Significance of Tumor Deposits in Stage III Colon Cancer. Ann. Surg. Oncol..

[63] Koelzer V.H., Zlobec I., Berger M.D., Cathomas G., Dawson H., Dirschmid K., Hädrich M., Inderbitzin D., Offner F., Puppa G. (2015). Tumor Budding in Colorectal Cancer Revisited: Results of a Multicenter Interobserver Study. Virchows Arch..

